# Supplementary material for: High-Protein Processed Foods: Impact on Diet, Nutritional Status, and Possible Effects on Health
Source: Nutrients. 2024 May 30;16(11):1697. doi: 10.3390/nu16111697 (PMC11174509; doi:10.3390/nu16111697)
Supplement: Supplementary file 1 [file nutrients-16-01697-s001.zip › nutrients-3022244-supplementary.pdf]

Supplementary Material

Survey about consumption patterns of high-protein processed foods

1. Gender:
    a. Woman
    b. Man
    c. Other
2. How old are you?
    a. 18-30
    b. 30-45
    c. 45-60
    d. > 60
3. Regarding physical activity, which group are you in?
    a. Light/sedentary
    b. Moderate/active
    c. High/intense
4. Do you know which foods naturally provide protein to your diet?
    a. Yes
    b. No
5. Do you know what protein-enriched products are?
    a. Yes
    b. No
6. If you know this type of products, could you say how you found out about them?
    a. Through ads on TV or on social media
    b. I saw them in the supermarket and decided to try them.
    c. Friends/family have told me about them.
    d. They have been recommended to me by a health professional (nurse, pharmacist, nutritionist, doctor…)
7. Regarding the following categories of PROTEIN ENRICHED products, which ones do you know?
    a. Dairy
    b. Jellies
    c. Energy bars
    d. Breakfast cereal: Muesli
    e. Bread
    f. Creams
    g. Snacks (chips…)
    h. Other option….
8. Have you ever consumed any protein-enriched products?
    a. Yes
    b. No
9. If the answer to the previous question is YES, please indicate which one(s):
    a. Dairy
    b. Jellies
    c. Energy bars
    d. Breakfast cereal: Muesli
    e. Bread
    f. Creams

    g. Snacks (chips…)
    h. Other option….
10. If you consume these types of products, indicate why:
    a. Because I think they are good for my health
    b. To increase my physical performance
    c. To shape my body
    d. It was recommended by a nutritionist/trainer/doctor…
    e. Other option….
11. If you consume these types of products, how often do you do it??
    a. One every day
    b. More than one a day
    c. I don't keep control, I consume them when I feel like it
12. Do you think these products are necessary for the general population?
    a. Yes
    b. No
13. Do you think these products are necessary for athletes?
    a. Yes
    b. No
14. Do you think that excessive consumption of this type of product can have a negative impact on health?
    a. Yes
    b. No
15. Do you consider that consuming these products brings any extra benefit to your diet?
    a. Yes
    b. No
